# Polymeric Materials with Antibacterial Activity: A Review

**DOI:** 10.3390/polym13040613

**Published:** 2021-02-18

**Authors:** Dania Olmos, Javier González-Benito

**Affiliations:** Department of Materials Science and Engineering and Chemical Engineering, Instituto de Química y Materiales Álvaro Alonso Barba (IQMAA), Universidad Carlos III de Madrid, Leganés, 28911 Madrid, Spain

**Keywords:** polymers, antibacterial, nanoparticles, biomedicine, food science

## Abstract

Infections caused by bacteria are one of the main causes of mortality in hospitals all over the world. Bacteria can grow on many different surfaces and when this occurs, and bacteria colonize a surface, biofilms are formed. In this context, one of the main concerns is biofilm formation on medical devices such as urinary catheters, cardiac valves, pacemakers or prothesis. The development of bacteria also occurs on materials used for food packaging, wearable electronics or the textile industry. In all these applications polymeric materials are usually present. Research and development of polymer-based antibacterial materials is crucial to avoid the proliferation of bacteria. In this paper, we present a review about polymeric materials with antibacterial materials. The main strategies to produce materials with antibacterial properties are presented, for instance, the incorporation of inorganic particles, micro or nanostructuration of the surfaces and antifouling strategies are considered. The antibacterial mechanism exerted in each case is discussed. Methods of materials preparation are examined, presenting the main advantages or disadvantages of each one based on their potential uses. Finally, a review of the main characterization techniques and methods used to study polymer based antibacterial materials is carried out, including the use of single force cell spectroscopy, contact angle measurements and surface roughness to evaluate the role of the physicochemical properties and the micro or nanostructure in antibacterial behavior of the materials.

## 1. Introduction

Infections caused by bacteria and other microorganisms lead to serious illnesses, septicemia and are the cause of many deaths every year worldwide [1,2]. Usually, to overcome an infection caused by bacteria, antibiotics are utilized as a general strategy. However, the excessive use of antibiotics has led to the development of multidrug resistant bacteria and consequently, stronger, or more complex formulations of antibiotics are necessary to fight them efficiently. One alternative to try to minimize the impact of this problem, is prevention, i.e., avoiding the proliferation of bacteria on different substrate surfaces by hindering their growth and development or simply preventing their adhesion. In this sense, research and development of novel antibacterial materials is becoming an excellent approach [3,4].

**Figure 1:**
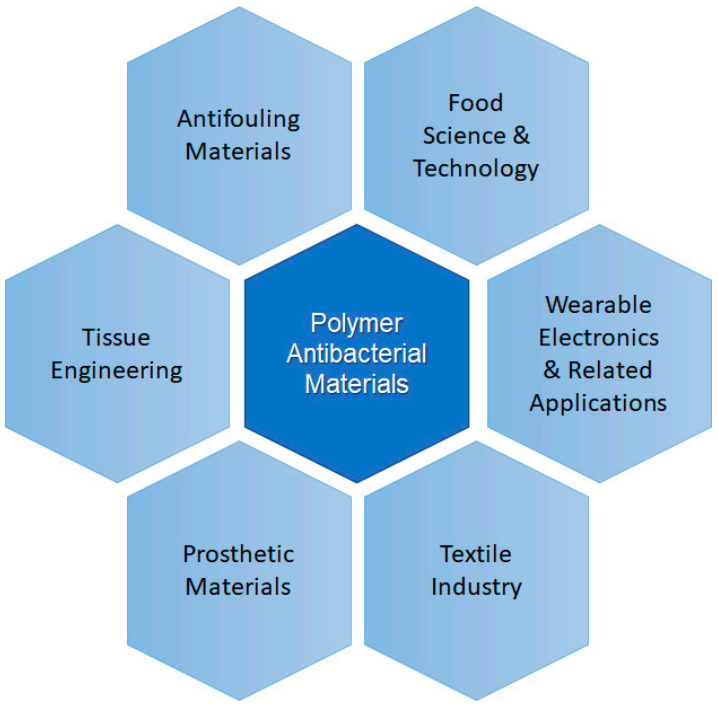
Scheme illustrating some areas of application of polymer antibacterial materials.

Polymers and polymer-based materials are present in many different applications, being those framed within the biomedicine field, which are perhaps recently receiving more attention [5,6]. Among the functions more desirable for materials used in medicine antibacterial action occupies one of the first positions, because bacterial growth could be prevented on medical devices, prosthetic materials, catheters (urinary or venous catheters) [7] and surgical masks [8]. Other areas in which polymer-based antibacterial materials are very useful and receive special attention includes food science and technology [9]. The use of antibacterial materials to prepare active or smart packaging to improve the food quality and extend its shelf-life is desired. Kuswandi reviewed the different desired functions of materials for food packing applications are presented [10]. The key functions of packaging materials are: (a) to serve as container of the product; (b) to preserve and protect the product, for example avoiding bacterial growth; (c) to present and sometimes to identify; and (d) to allow transportation and distribution of the product and to provide information of the product to the consumers. In this review, we will focus on strategies to avoid food spoilage. Apart from these two clear examples, the use of polymeric materials with antibacterial properties is present in many other applications related to electric polymeric materials or the textile industry. In Figure 1, a scheme illustrating the different applications that will be considered in this review is presented. 

**Figure 2:**
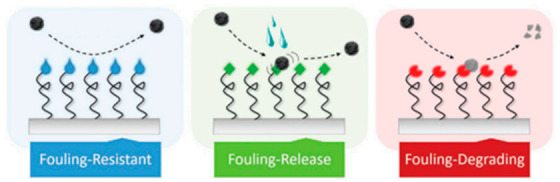
Illustration of the main antifouling strategies, as described by Kamperman et al. (Figure reproduced with permission from Reference [19]).

In this work, the main uses of polymeric materials in the areas presented in Figure 1 will be addressed. The review is focused on the preparation and characterization of polymeric systems based on neat polymers or modified with a filler, usually nanoparticles that provide the functionality to the overall material. Among the different particles used silver (Ag), copper (Cu), titania dioxide (TiO_2_), zinc oxide (ZnO) are well-known examples [11,12,13,14,15,16]. In addition to compositional factors, the use of different processing strategies is also presented here as an alternative to obtain antibacterial materials. One common approach to lower bacterial adhesion is via nanostructured or nanopatterned surfaces to produce antifouling materials, either via the chemical modification of surfaces or via nanopatterning [17,18]. In Figure 2, a schematic illustration of the main antifouling mechanisms is presented. According to Kamperman et al. [19], the antifouling action of the surfaces can be due to a fouling resistance surface, or to a surface that lowers the interaction between the foulant agent and the surface (Fouling release) or chemically degrading or killing the flocculants (fouling–degrading).

The aim of this paper is to bring readers attention on the broad spectrum of the uses and applications of polymeric materials with antibacterial properties and the need for continuous research in this field. First, a revision of some common uses of antibacterial polymer-based materials is presented, highlighting the application and the antibacterial mechanisms. Usually, most antibacterial materials based on the action of different particles (Ag, Cu, TiO_2_, ZnO) or polymers (chitosan) imply the formation of reactive oxygen species (ROS) that alter cell metabolism causing its death (biocide action). However, other effects ascribed to the inhibition of bacterial adhesion must also be considered. Then, a revision of the main methods of preparation with their advantages and disadvantages is presented. Finally, an overview of the main characterization techniques is given, paying special attention to the study of the antibacterial action.

## 2. Polymeric Materials with Antibacterial Activity

### 2.1. Antibacterial Materials for Biomedical Applications

The presence of antibacterial materials in biomedicine and related fields of science and technology has become essential. Every year, a lot of people die due to infections caused by pathogens [3]. Among the different pathogens (bacteria, virus, fungi, algae, and others), pharmaceutical industry is paying special attention to bacteria, mainly due to the appearance of what is called multidrug resistant bacteria (MDR). According to the World Health Organization (WHO), resistance to antibiotics is one of the biggest dangers to health and food security worldwide, as antibiotic resistance can affect anyone of any age and in any place of the world. The most frequent bacteria are *Acinetobacter*, *Pseudomonas* and some enterobacteria such as *Klebsiella, E. Coli, Serratia* and *Proteus*. These bacteria can cause serious infections such as bloodstream infections and pneumonia, and even death.

**Figure 3:**
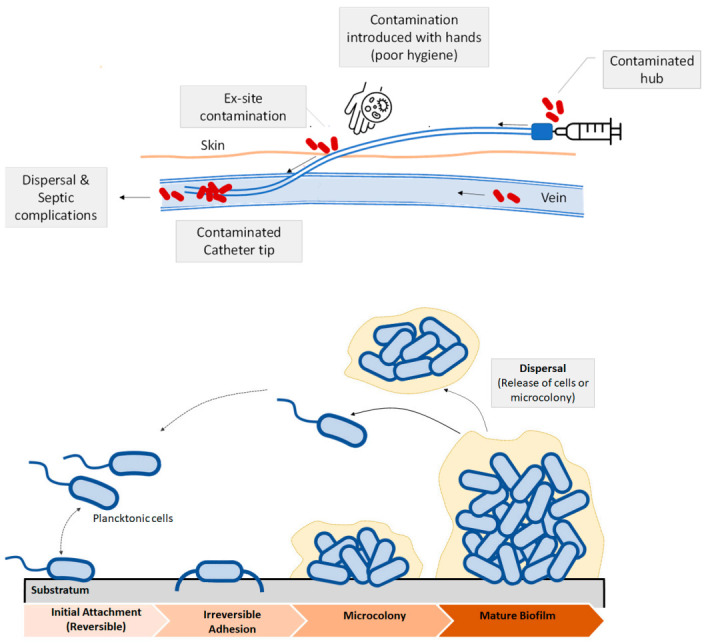
(**Top**) Schematic view of biofilm formation in different regions of a venous catheter. Microbial contamination may come from the catheter hub, the patient’s skin, due to the lack of skin antisepsis or from the bloodstream. (**Bottom**): Biofilm development and dispersal of bacteria or microcolonies.

Bacterial infections related with implants and medical devices are generally known as device related infections [11]. Bacterial growth and development on the surface of biomedical devices and implants has been regarded as the main cause of device related infections (DRI) [11,20]. Some recent studies that consider the impact of infections due to bacterial colonization in medical devices reported that urinary catheter infections and central venous infections were the most frequent ones, followed by orthopedic implants. Catheter-associated urinary tract infection (CAUTI) is one of the most frequent infections developed in hospitals [21]. The catheter is colonized by bacteria or pathogen agents thus causing biofilm formation, which increases the probability of developing secondary bloodstream infections. Microbes or bacteria can enter via the catheter hub or from the patient’s skin. Contamination can lead to the formation of a colony, and then, to a mature biofilm. Apart from catheters other device-related infections are observed in prosthetic cardiac valves, pacemakers, vascular grafts, orthopedic implants or prosthetic joints and contact lenses as some examples [7]. Post-operative infections due to orthopedic implants are also common [22,23]. Methicillin resistant *Staphylococcus Aureous* (MRSA) and *Escherichia Coli* are the major pathogens in medical device related infections [24]. One of the most common strategies to fight these bacteria is based on the use of controlled release drugs or antibiotics. This approach has some evident benefits as it inhibits biofilm formation, reduces the risks of suffering infections, thus lowering patient’s mortality. However, the use of broad-spectrum antibiotics may increase the risk of developing multidrug resistant bacteria [25], as well as causing systemic toxicity [26]. In Figure 3, a scheme illustrating the microbial contamination of a catheter is shown. Microbes or bacteria can enter via the catheter hub or from the patient’s skin (Figure 3, top). Contamination can lead to the formation of a colony, and then, to a mature biofilm. Once the bacteria adhere irreversibly, a microcolony and then to a mature biofilm occurs (Figure 3, bottom). 

**Figure 4:**
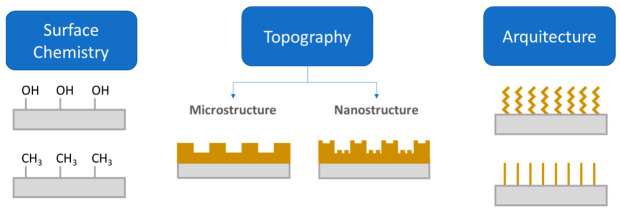
Illustration on three approaches to endow a surface with antifouling properties: (1) modification of surface chemistry; (2) Surface topography; and (3) the architecture of the coating (Figure adapted with permission from Kamperman et al. [19]).

Different approaches have been used to address the problem of device-medical infections. Apart from the treatment with antibiotics, one common strategy is the use of antifouling or antimicrobial coatings. According to Francolini and collaborators [27], antifouling coatings can be prepared from (i) polyethylene glycol; (ii) enzyme or zwitterionic based coatings and (iii) superhydrophobic coatings. Functionalization of surfaces with polyethylene glycol (PEG) or oligo (ethylene glycol) imparts adhesion resistance to proteins and other biological agents [28]. Steric hindrance caused by physically adsorbed or chemically covalently bonded PEG chains usually reduce protein adsorption, thus limiting the adhesion of protein or other biomolecules [28]. In a recent work, Hendrick, Yang and coworkers [29] developed a coating that allowed us to obtain antibacterial and antifouling surfaces in catheter surfaces using a facile method in a single step. Brush-like structures based on polycarbonates with pendant adhesive dopamine, polyethylene glycol and antibacterial cations were proven to provide a long-term treatment that is effective against *S. Aureus* and *E. Coli*. However, not only the chemical modification accounts for producing antifouling materials, but also the surface micro or nanostructure and the surface architecture. All these factors are illustrated in Figure 4. 

The effect of surface architecture in antifouling/antibacterial behavior of triblock copolymers is discussed in several works. Zhou and collaborators [30] revised the different strategies to fabricate brushes on a wide variety of surfaces, either from physical adsorption or by chemical coupling reactions, grafting copolymers by surface initiated radical polymerization (SIRP). In different research, Yang et al. [31] focused on the study of antibacterial properties vs. the blockcopolymer structure. The copolymers consisted of three blocks, an antifouling PEG, antibacterial cationic polycarbonate, and maleimide-functionalized polycarbonate (so that the blocks could be anchored on the surface of a silicone rubber, like those used for a catheter tube). It was observed that the block’s topology affected the antifouling properties of the coatings. The surfaces coated with the tethering block (2.4k-V), so-called V-shaped copolymer, did not prevent bacteria fouling on the surfaces, whereas the surfaces coated with the 2.4k-S, called S-shaped copolymers, did not present fouling. The S-shaped copolymers were effective against *S. aureus* and *E. Coli* after 1 week of culture in each case. The results from this research proved that blocks topology highly affects the final antifouling behavior. Furthermore, the coatings did not present adverse effects against blood, showing its potential applications as antifouling coatings for intravenous catheters.

Another field of recent interest in biomedicine is the field of Tissue Engineering (TE). The growing need for organs has led researchers to consider the reconstruction of organs and tissues, leading to the birth of a discipline known today as Tissue Engineering [32]. According to *Langer* and *Vacanti* [33], tissue engineering is an interdisciplinary field that applies the principles of *engineering* and life *sciences* towards the development of biological substitutes that restore, maintain or improve the function of a biological tissue or a complete organ. The goal is not to perform an organ transplant, but to find the right way, the body itself can integrate, into its structure, a tissue manufactured in the laboratory to facilitate recovering the functionality of the damaged organ or tissue. From the point of view of Materials Science and Engineering, the design of functional materials that can be used as scaffolds for TE is especially important. Tissue related infections involved chronic otitis, sinusitis, endocarditis, lung infections, biliary tract infections, urinary tract, osteomielitys or chronic wounds [7].

A broad variety of polymers, either natural or synthetic polymers or combinations of both can be used [34,35]. Apart from the inherent biocompatible character of the scaffolds, another desirable characteristic is their antibacterial or antimicrobial properties to prevent biofilm formation during the growth of the new tissue. Polymers usually do not have intrinsic antibacterial properties, except chitosan, which has been proven to present antibacterial properties [36,37,38,39,40]. To prepare polymer antibacterial materials, antimicrobial or antibacterial additives are used to modify the polymer matrix using different methods. Among the most widely used systems, those based on biodegradable and biocompatible polymers filled with antibacterial particles are preferred, for instance, those based on polyhydroxyalkanoates such as *polylactic acid (PLA)* [41,42,43], *polyglycolic acid (PGA)* and *polyhydroxybutyrate (PHB)*.

**Figure 5:**
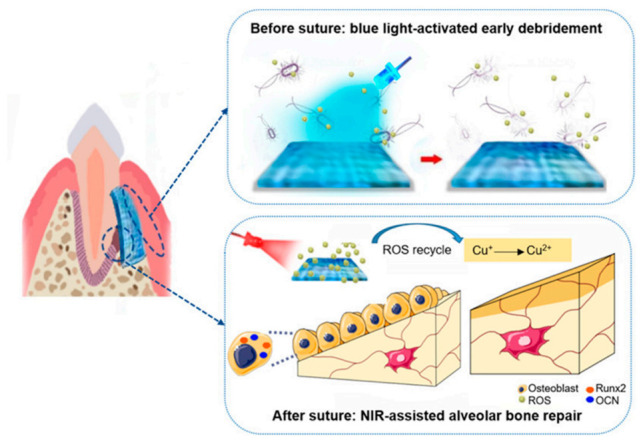
Jelly-inspired injectable hydrogel composite materials for guided tissue regeneration strategies. Before suture, under blue light excitation, sodium alginate hydrogel composite (CTP-SA) was activated to produce ROS to kill bacteria, achieving early debridement. After suture, under NIR irradiation, the photothermal effect of CTP-SA via the ROS recycle promote the osteogenesis (Reprinted with permission from Yingying Xu, Siyu Zhao, Zhenzhen Weng, Wei Zhang, Xinyi Wan, Tongcan Cui, Jing Ye, Lan Liao, and Xiaolei Wang ACS Applied Materials and Interfaces 2020 12 (49), 54497–54506 DOI: 10.1021/acsami.0c18070. Copyright 2020) [45].

Multifunctional component materials are usually required for TE applications. For example, a polymeric periodontal membrane with antibacterial and osteogenic properties was developed [44]. In this work, osteoconductive, antibacterial, and flexible films of poly(caprolactone) (PCL) filled with zinc oxide (ZnO) nanoparticles were prepared by electrospinning. Porous films with controlled mechanical properties were prepared for the treatment of periodontitis via guided tissue regeneration (GTR). The PLC/ZnO membrane is placed between the gum flap and the alveolar bone in a defect created on a rat model. The addition of ZnO improved osteoconductivity of the periodontal membrane, as well as its antibacterial activity. In Figure 5 [45], a jelly based polymer is used as a strategy for a guided tissue regeneration strategy to treat periodontitis, a bacterial infectious disease that can cause the loss of tissues supporting teeth. X. Wang et al. [45] proposed the use of a hydrogel based on cuprous oxide (Cu_2_O) and polydopamine coated titanium dioxide nanoparticles as a strategy to produce a new tissue using a film such as that shown in Figure 5. 

Thermosensitive hydrogels based on chitosan modified with beta-glycerophosphate (β-GP) were prepared to treat defects in bones [46]. These materials are gaining more attention in the field of bone tissue engineering (BTE) because of their ability to successfully regenerate bone tissue without the need for surgical intervention, thus lowering the invasiveness of the treatment [46]. Other interesting kinds of materials are those developed by Tamayol et al. [47]. In this work, a system based on poly(glycerol sebacate) (PGS) and poly(ε-caprolactone) (PCL) filled with calcium peroxide was developed, thus producing materials with the ability to release oxygen. The lack of oxygen can lower the growth rate of the tissue because it causes a hypoxic environment for the cells. The development of scaffolds with the ability to release oxygen increased metabolic activity of the cells favoring the development of the new tissue. Additionally, silk fibroin scaffolds have been reported for bone tissue engineering [48]. Silk fibroin is a natural protein with good mechanical properties and which offers the ability to tune its degradation rate.

Among the different tissues, vast research has been carried out in bone tissue engineering (BTE), due to the lack of suitable autografts or allografts. However, extensive research in the field of TE is being done and a large variety of studies have been published for other tissues such as skin, heart or kidney. Research on skin, due to wounds caused in burns is also another area of interest. Natural polymers like chitosan, hyaluronic acid (HA), collagen or gelatin and synthetic polymers like polyethyleneglycol (PEG) or polylactic-co-glycolic acid (PLGA) are present in studies related to skin tissue engineering [49,50,51]. Chitosan materials filled with selenium nanoparticles were proven to be effective against infections in burnt wounds [52]. Additionally, scaffolds based on silk fibroin-collagen loaded with titania nanoparticles were adequate for skin tissue regeneration [53]. Polyuretanes, due to their wide range of mechanical properties, varying from flexible to rigid materials have been widely used in many industrial and biomedical applications [5]. Hybrid polyurethane-polylactide porous scaffolds modified with ciprofloxacin were prepared as an antibacterial scaffold for skin regeneration applications [51].

**Figure 6:**
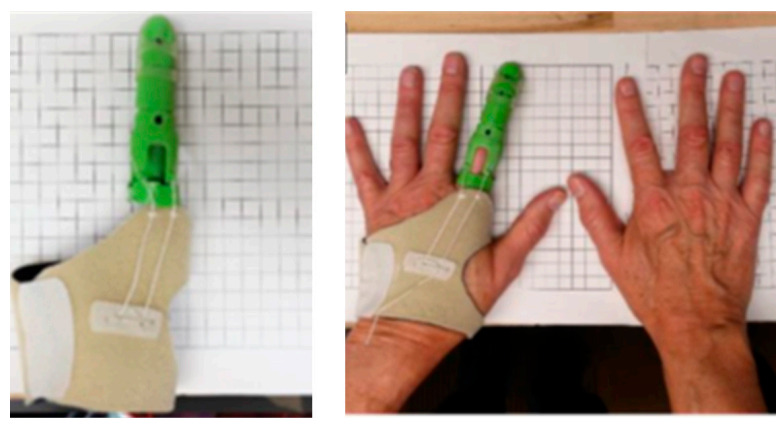
Examples of 3D printed antibacterial prosthetic material with a commercial filament of PLA with 1–3% copper nanoparticles, PLACTIVE^TM^ (Image reproduced with permission from [54]).

With the advent of 3D printing or additive manufacture, the field of TE has experienced an increase in the preparation of different kind of scaffolds. For example, Li and collegeues prepared a 3D printed structure using PLA filled with nano-hydroxyapatite or modified with polydopamine for applications in BTE [42], although many other examples can be found in literature. Apart from the application of 3D printed materials as scaffolds, another interesting application is their use as external prosthesis or for the preparation of medical devices like surgical instruments with antibacterial or antimicrobial properties at a low cost [54]. In a brief report, J.M. Zuniga showed a real example based on the preparation of a prosthetic material with a commercial antibacterial PLA filament, PLACTIVE^TM^. The antibacterial properties were obtained due to the addition of small amounts of copper particles (1–3%). In Figure 6, some examples of 3D printed medical devices and 3D printed prosthesis developed by. 

### 2.2. Antibacterial Materials in Food Industry

**Figure 7:**
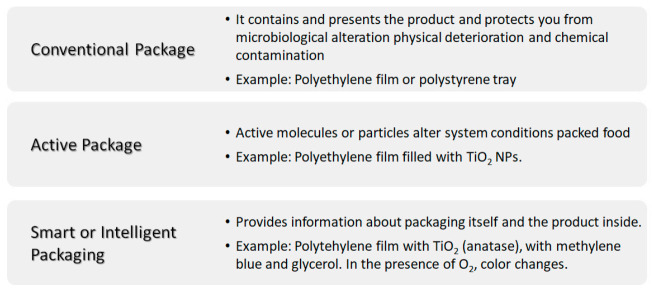
Scheme summarizing the different kinds of packaging: conventional, active and smart or intelligent packaging.

Food quality and food security are some of the biggest concerns within the food sector or food industry. Food storage is a key factor to prevent disease transmission. In this sense, the materials used in food packaging play an important role in both the food and health sectors. From the point of view of the food packaging industry there are different possible approaches when using materials (Figure 7): (i) *conventional packaging*, where the container acts as a passive barrier separating the content of the package from the environment; (ii) *active packaging*, where the material used coordinates with food to improve food safety and quality extending its shelf life and (iii) *smart packaging* or *intelligent packaging*, apart from protecting the content from the environment, it responds to possible changes of food or food environment with the possibility of providing information about the package itself and/or the product inside it [55]. The so-called nanosensors in smart packaging, respond to stimuli caused by changes in the immediate environment such temperature and humidity variations, oxygen concentration or the presence of degradation byproducts [55,56]. For example, oxygen indicators are prepared with titania dioxide nanoparticle (anatase) in the presence of glycerol, an electron donor, and methylene blue. Other oxygen sensors based on nanoparticles of SnO_2_ have also been developed; however, TiO_2_ is biologically inert, whereas SnO_2_ needs further investigation, as it can have adverse effects on skin, eyes or if swallowed [57].

Two examples of widely used active packaging are packaging with antimicrobial agents and systems based on the controlled release of active substances [58]. In controlled release containers, an active substance incorporated into the packaging material, usually an antioxidant or food preservative, is gradually dispensed in the food to prevent its deterioration. However, antimicrobial packaging refers to the direct incorporation of antimicrobial substances into the packaging material. The usual goal in both cases is to prevent the growth of bacteria and the formation of biofilms on the surface of food, which is where the degradation process usually begins. This is a very important concern because only a few bacterial cells surviving in food is enough to cause very serious diseases [59].

Different natural or synthetic polymers can be used for packaging in the food industry. A vast number of articles and reviews are available in the literature devoted to the preparation and characterization of food packaging antibacterial or antimicrobial materials [2,60,61]. Among the different kinds of particles used to confer antibacterial properties to polymers, silver, copper, TiO_2_, and ZnO have been extensively used. Antimicrobial activity of silver nanocomposites is well-known. Silver particles and silver nanoparticles (AgNPs) are frequently added to prevent the growth of different microorganisms. AgNPs are effective against food borne pathogens such as *E. Coli* [62], *L. Monocytogenesis* [63,64], and *S. Aureus*, *Pseudomona Fluorescens*.

The mechanism of action of silver and silver nanoparticles is still discussed, but the most accepted one considers silver ions (Ag^+^) release from silver nanoparticles (Ag NPs). Ag+ ions can interact with the proteins in bacteria, for example via formation of S-Ag bonds with thiol groups present in cysteine and other compounds, interrupting the electron transport chain and avoiding the ability to replicate DNA. Additionally, the presence of silver ions in solution (Ag+) can modify osmotic pressure, inducing the release of intracellular material, which can lead to subsequent cell membrane collapse. Hsieh and colleagues [65] measured, as a function of time, the release in the solution of silver ions from dopamine/Ag modified surfaces, and although the concentrations are very small (~2755.5 ± 3.8 µg L^−1^) of Ag^+^ after 3 h, it is probably enough, at least locally, to change the osmotic pressure to finally prevent bacterial adhesion. Results of that work revealed that silver action is effective against bacteria and safe with endothelial cells, showing their potential use in catheters. Silver has been effectively used in different polymers for food packaging applications, such as polypropylene, low density polyethylene [66,67], poyhydroxibutyrate (PHB) [68] or EVA [69], PS.

Copper nanoparticles [70] and copper oxide [71] have also been used for the preparation of polymer-based materials in food science applications. According to Sun et al. [11] and other researchers, there are different potential bactericide mechanisms of copper: (i) oxidative stress of cells due to high levels of copper; (ii) changes in osmotic pressure, leading to a leakage of magnesium and other cell nutrients, and (iii) unspecific binding of copper to other proteins, thus producing a failure in the functionality of the proteins. Apart from the use of chitosan as a polymer matrix, chitosan is also used to produce hybrid nanoparticles in combination with silver or copper nanoparticles. The antibacterial action of chitosan is attributed to a change in the permeability of the cell wall, causing its rupture and thus bacterial death. It usually implies the interaction between positively charged chitosan and cell membranes, negatively charged, causing an increase in the permeability of the membranes, which provokes cell dead [60].

Photocatalitic activity of titanium dioxide (TiO_2_) and ZnO is used to prepare active coatings and polymer nanocomposite materials. Photocatalyst promotes the oxidation of polyunsaturated phospholipids of microbial cell membranes, inhibiting the proliferation of microorganisms [55]. In any case, the combination of silver with titania nanoparticles seems to enhance the antibacterial properties of materials [72]. The mechanism of action of ZnO is similar to that of TiO_2_. In the presence of an electromagnetic radiation (light with enough energy) the electrons and holes react with adjacent molecules (O_2_, H_2_O, for example, thus leading to the generation of ROS. Some studies reported that the creation of ROS species can occur even under dark conditions [73]. For the different antibacterial agents, the ROS generation plays an important role in antibacterial properties, as it damages cells from DNA deterioration, resulting in the cell death. In a recent review on the mechanisms of antibacterial properties of ZnO nanoparticles [74], it is proposed that the generation of the ROS occurs via the interaction of ZnO with other molecules present in solution, such as oxygen or water, according to the following reactions:

ZnO Nps • → e^−^ O_2_ + O_2_^•−^ (1)

O_2_^•^^−^ + H_2_O → ^•^HO_2_ + OH^−^^•^HO_2_ + ^•^HO_2_ → H_2_O_2_ + O_2_ (2)

H_2_O_2_ + O_2_^•−^ → O_2_ + ^•^HO + OH^−^ (3)

Carbon nanotubes (CNTs) have also been reported to be effective against *E. Coli*. One possible interpretation is the fact that the high aspect ratio of the long and thin nanotubes allows for puncturing of microbial cells, thus causing irreversible damages and bacterial death [55]. In addition, antibacterial properties of single walled carbon nanotubes (SWCNT) are more marked than in multiwalled carbon nanotubes (MWCNTs). In a recent review, the different antibacterial properties of carbon nanomaterials are discussed [75]. Additionally, in a recent study on low density polyethylene, LDPE, filled with MWCNTs [76], a correlation between hydrophobicity, biofilm development and the shape and size of DH5α *E. Coli* was observed, indicating that the presence of MWCNTs leads to an antibacterial effect by decreasing cells adhesion and changing their metabolism.

### 2.3. Antibacterial Polymer Materials in Electric and Electronic Aplications

In the last decades, research on conductive self-healing hydrogels has increased considerably [77,78,79,80,81]. The interest in this family of polymeric materials lies in their versatility and their potential uses in a wide variety of applications such as electronic skin, wound healing, human motion sensors, self-repairing circuits, soft robots, biomimetic prostheses and health monitoring systems [82]. In a recent review, B. Guo and colleagues [83] summarized some of the most recent applications of these materials.

P.X. Ma and coworkers [81] have reported the use of self-healing conductive hydrogels biodegradable and injectable with potential uses in cardiac cell therapy. The hydrogels were based on chitosan-graft-aniline tetramer (CS-AT) and dibenzaldehyde-terminated poly(ethylene glycol) (PEG-DA). The hydrogels were injectable and had good adhesion properties to host tissue (cardiac cells) and a conductivity similar to that of the native cardiac tissue (10^−3^ S·cm^−1^) as well as antibacterial properties. In another work, P.X. Ma and colleagues [80] reported the use of conductive self-healing hydrogels for wound healing applications. These hydrogels were based on quaternized chitosan-g-polyaniline (QCSP) and benzaldehyde group functionalized poly (ethylene glycol)-co-poly(glycerol sebacate) (PEGS-FA). It was found that hydrogels with a crosslinker concentration of 1.5%, wt% were optimal for wound healing in terms of in vivo blood clotting.

M. Xing and cols. [82] prepared ultrastretchable skin-inspired multifunctional hydrogels based on poly(acrylic acid) and ferric ions, as part of the dynamic ionic interactions, together with a conductive polymer network of polypyrrole. The self-healing hydrogels had presented some interesting properties such as electrical conductivity, electrical and mechanical self-healing properties with a 100% mechanical recovery in 2 min, as well ultrastretchability, with a 1500% elongation, and a good response as a pressure sensor. Additionally, a polydopamine-based hydrogel inspired by animal skin was prepared in a recent work [84] for epidermal sensors and diabetic foot wound dressings. The hydrogel fabricated from polydopamine with silver nanoparticles (AgNPs), polyaniline and polyvilnylalcohol had many attractive properties. Apart from the mechanical and electrical tunable response, the conductive self-healing hydrogel had a remarkable response on diabetic foot wounds, favoring angiogenesis and collagen deposition, as well as inhibiting bacterial growth and biofilm formation (*E. Coli* and *S. Aureus*). These materials also present high self-adhesion to different biological tissues such as heart, spleen, lung, liver and skin [84].

**Figure 8:**
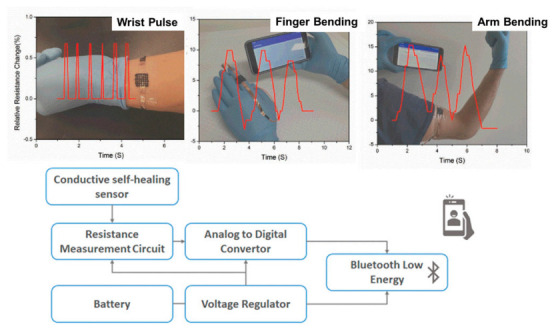
(**Top**): Examples of sensors used to measure wrist pulse, finger bending and arm bending. (**Bottom**): Block diagram of the wireless bodily motion detection system (this figure was adapted with permission from the authors in Reference [82]).

In wearable electronics, the use of self-healing hydrogels has shown promising results. In some recent works, flexible wearable electronic devices were studied [85]. Polymer nanocomposites based on N-isoprobpyl acrylamide filled with nanoclay (laponite) and MWCNTs were examined. Pressure dependence conductivity of the nanocomposites was analyzed by probing their potential applications for human motion sensors (pulse detection, elbow or knee bending, finger bending, Figure 8). Low-cost flexible and wearable antibacterial piezoresistive materials were prepared by depositing carbon nanotubes and polypyrrole coating on a conventional polyurethane elastomer, PPy/CNT/PU [86]. The PPy/CNT/PU composites had an antibacterial activity against *S. Aureus, E. Coli* and *K. Pneumoniae*, which was explained in terms of a similar mechanism of diffusion of anionic species and active nitrogen of PPy to that of quaternary ammonium salts. An antibacterial synergistic effect was obtained from the combination of the diffusion mechanism with the lysing of the microbial cell walls due to the presence of CNTs. 

In a recent study, Ren et al. [87] developed a novel strategy to produce medical devices with antifouling and antibacterial properties. The work proposed the preparation of PDMS filled with superparamagntetic Fe_3_O_4_ particles, which can be remotely controlled [87]. Additionally, Poly(vinylidene fluoride), PVDF and its copolymers are piezoelectric materials with good physical and mechanical properties [88]. The piezoelectric response of this polymer depends on the crystalline phases, whereas the morphology and mechanical properties depend on its crystallinity [88,89,90,91]. A good correlation in piezoelectric response was obtained for PVDF with low loadings of AgNPs (0.4%), showing their potential for the design of self-powering devices used as nano-generators with antibacterial properties.

**Figure 9:**
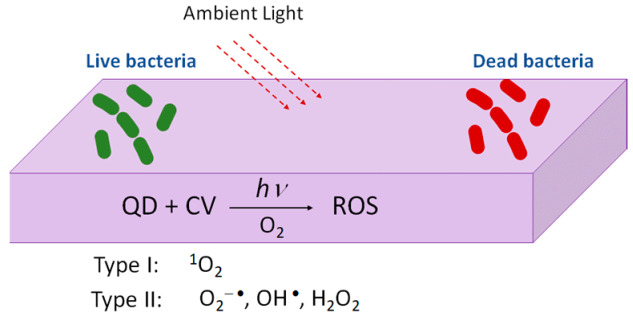
Figure illustrating the mechanism of photoactivable polymers with visible light combined with QDs + CV.

The interest in quantum dots (QDs) for photobacteridical applications relies on the incidence of antibiotic resistant infections arising from contaminated surfaces in hospitals or due to device related infections (DRIs). Under the exposure to ambient light, photoactivable surfaces can generate reactive oxygen species [92,93] (Figure 9). Complexes of Quantum Dots (QDs) and crystal violet (CV) were formed via the absorption of the CV. These complexes (QDs + CV) were then dispersed in a medical grade polyurethane. The results showed that the QDs-CV complexes had a good response against antibacterial activity in *MRSA* and *E. Coli*. The exact antibacterial mechanism of action of QD + CV is not totally understood yet. It is suggested that there are two pathways, known as Type I and Type II mechanisms. As proposed by Owusu and cols [94,95] Type I mechanism involves photo-electron transfer (PET) to generate free radicals such as superoxide anions and hydroxyl radicals. The Type II mechanism involves the formation of reactive singlet oxygen (^1^O_2_) from a direct energy transfer from the phtosensitizer to molecular oxygen. Both mechanisms contribute to the formation of ROS that kill bacteria. As the purpose of this study is the use of these complexes in medicine, cadmium free quantum dots were proposed [94]. The photoactivable mechanism is also proposed in a household solar water disinfection device, where self-supported TiO_2_ is placed inside polyethylene terephthalate (PET) bottles to produce clean household water [95].

### 2.4. Antibacterial Polymers in Textile Industry

The interest in preventing proliferation of microbes and bacteria on textiles during its use and storage has increased the research in this area. Textiles used in sports, medicine and daily life are modified either incorporating the antibacterial agent in the textile fiber during processing or using antimicrobial finishing treatments. Most common active agents used in textile industry include silver [18,96,97], ZnO [98,99], chitosan [100,101,102], quaternary ammonium salts [102], polypyrrole [103], triclosan [104], dyes and regenerable N-halamine compounds [105] and peroxyacids.

Among natural fibers cotton fibers are probably the most widely used. Chen [106] and Xin [107] reported an alternative approach to produce stable antimicrobial cotton fibers. The paper reports the finishing process of cotton fibers with reactive siloxane sulfopropylbetaine (SSPB). The finishing agent SSPB contains sulfopropylbetaine groups and reactive siloxane groups that can be covalently bound on the surface of the cotton textile. The SSPB is well immobilized due to the covalent bonds formed with the cotton fibers and showed good antimicrobial response against *S. Aureus* and *E. Coli*, thus resulting in a durable and non-leaching antibacterial fiber. Zhu and colleagues [108] chemically bound poly(hexamethylene guanidine) to the surface of cotton fibers obtaining antibacterial cotton fibers with antibacterial activity against *Escherichia Coli* and *Staphylococcus Aureus* was maintained even after 1000 consecutive washes in distilled water.

**Figure 10:**
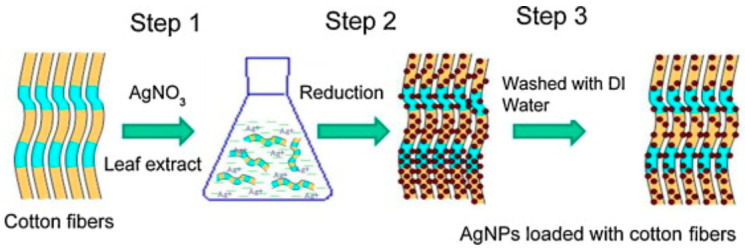
Illustration of AgNPs loaded cotton fibers via a three-step method. The leaching out with water leads to a decrease in the number of particles due to poor binding (Reproduced with permission from Reference [113]).

Silver have been widely used in textile industry and for this reason different approaches to synthesize silver nanoparticles using green methods have been used [68,109,110,111]. For example, it is interesting to highlight the biosynthesis of silver nanoparticle using aqueous solutions of bamboo leaves extract [109] yielding almost spherical silver nanoparticles. The phenolic acids and other flavonoids are used as bio-reductant agents to produce the nanoparticles, avoiding the use of more hazardous chemicals [112]. In another research, antimicrobial cotton fibers were loaded with Ag NPs and obtained and deposited via a simple process [113], as illustrated in Figure 10. The poor binding of AgNPs decreased the amount of particles in the fibers.

## 3. Preparation Methods

Most polymeric materials with antibacterial properties are based on the combination of one or two polymers generally modified by the addition of inorganic particles or active molecules that can inhibit bacterial growth. These active molecules can be mixed with the polymer or added afterwards as a post-treatment or finishing treatment. For this reason, most processing methods that are used in polymers can be used to prepare polymeric materials with antibacterial properties.

Melt processing is a quite common method to process thermoplastic polymers. This method is easy to use, adaptable to different systems and conditions and it is good for large scale productions, which explains its widespread use in the industry (melt mixing, extrusion, and injection molding) [114,115,116,117]. Melt processing is suitable to prepare solid bulk pieces of materials of either pure polymers or of polymer filled composites, being possible to disperse the particles with the antibacterial properties, such as AgNPs within the polymer matrix. This method may not be adequate for processing systems modified with organic molecules such as antibiotics, some antioxidants or any other organic molecules sensitive to temperature. For example, B. Kaffashi and coworkers [114] optimized the processing conditions to prepare a bionanocomposite based on poly(ε-caprolactone) filled with polylactic acid (PLA) nanoparticles loaded with triclosan (TC). The samples were prepared mixing the particles with the PCL in a Brabender 50 EHT mixer and then molding them in a hot-press at 120 ºC. The processing temperature was adjusted to avoid melting of the PLA/TC particles and to favor a prolonged action of the drug release. In addition, the compositional and rheological properties of these samples were suitable to process them by melt spinning, which is an option to prepare materials constituted by fibers leading to interesting morphologies for tissue engineering applications [118].

In a recent work, Almeida Neto and cols [116] prepared a pre-mixture of poly(3-hydroxybutyrate-co-3-6%hydroxyvalerate) (PHBV), nanodiamond (nD) and nanohydroxyapatite (nHA) loaded with vancomycin (VC). The components were mixed in solution, then subjected to a process of solvent evaporation (rotatory evaporator) and then injection molded to prepare specimens for the corresponding characterization. This formulation combines bioactive biodegradable materials with a release drug delivery action, promising for the treatment and prophylaxis of bone infection.

Processing of composites may also involve the dissolution of the polymer and the dispersion of the inorganic particles or antibacterial particles in the solvent. Other common approaches to prepare polymers and polymer composite materials are from solution. Perhaps, the simplest case is solvent casting [119,120,121,122,123]. A solution of the polymer in a volatile solvent is prepared and poured in a Petri dish, letting the solvent evaporate and obtain a polymer film. This method is not easily scalable to the industry and when particles are big enough, compositional gradients can be obtained due to gravity action. In some cases, to obtain homogeneous distribution of the particles across the thickness of the sample, casted films can be post-processed by applying a hot-pressing treatment [124]. Dipping or immersion methods are other alternatives in which polymer solutions are used and may be suitable for applying a surface coating or a finishing treatment [125,126]. In polyolefins, such as polyethylene or polypropylene, widely used in industry, this approach is not very recommended as they are poorly soluble in most solvents. Polyolefins are mainly soluble in benzene or xylene derivatives and using high temperatures. Besides, the toxicity of these solvents is high, and because of this, other processing alternatives are used for polyolefins such as melt compounding [66] or surface treatment via plasma [127,128].

Other methods that imply the use of polymer solutions are electrospinning and solution blow spinning. In electrospinning, a solution of the polymer or the polymer mixed with a suspension of the particles is drawn in the presence of an electric field, leading to the formation of fibers with diameters that vary from various nanometers to a few hundreds of nanometers or submicrometric size. In solution blow spinning, a solution is ejected from a nozzle by a propulsion gas and ejected into a collector. Both electrospinning and solution blow spinning can be scaled up. In fact, electrospinning has been successfully used to a wide variety of applications in the textile industry [129,130], membranes [131], pharmaceutical industry [132], and for biomedical applications, such as scaffolds in TE [44,133] or wound dressings [134]. Solution blow spinning has been proven to be a good alternative method and versatile as it can be done on any surface and is faster, as compared to electrospinning. Morphologies of the porous materials are less homogeneous in terms of the diameter of fibers produced than in electrospinning. Both electrospinning and solution blow spinning allow obtaining variable morphologies, including corpuscles or beads, fibers or a combined mixture of corpuscles plus beads, depending on the viscosity of the solution and the processing conditions [91,135,136,137,138] that can condition the wettability behavior and particle adhesion such as cell adhesion [139].

Solid state methods such as high energy ball milling (HEBM) have been successfully used to prepare polymer nanocomposites, reaching uniform dispersion of the particles even for relatively high loadings [140,141,142,143]. In general, HEBM has been used for the mechanical alloying of metallic or ceramic materials, but at the beginning of the 20^th^ century, it was first used for polymers by Castricum [144]. Since then, some interesting works in the field of polymers, polymer blends and polymer based composite materials have been published, including investigations focused on the food industry applications, polymers for electric and electronic applications, some of which are already collected in a review [142,143,145,146]. From the point of view of processing methods, HEBM provides an alternative to efficiently disperse particles without using chemical methods to modify the particles or the polymer matrix and avoiding the use of solvents. The main drawbacks are that due to the high energy transfer, chain scission or crosslinking mechanisms may occur. Another important point that should be taken into consideration is that cross contamination due to HEBM may occur, although it is usually very low; for instance, less than 0.03% wt/wt of Fe, determined by atomic absorption spectroscopy (AAS), when using stainless steel as milling tools [146]. Therefore, this method is suitable for preparing materials for food packaging applications. However, although the amount of iron detected is small, this fact should be considered for applications related to some areas of medicine.

Polymeric materials for active food packaging applications have been prepared by milling. For example, biodegradable polymer composites based on pectin and organically modified montmorillonite were prepared by Vitoria and coworkers [140]. LDPE polymer nanocomposites filled with titania (TiO_2_) [147] or silver (AgNPs) [148] were also prepared. In particular, a decrease in the number of colony forming units (CFU) of *Pseudomonas fluorescens* and changes in the aspect ratio of DH5 α *E. Coli* bacteria were reported in the system LDPE/TiO_2_ prepared by HEBM. These results indicate that HEBM might be used to produce antimicrobial or antibacterial polymers with potential applications in the health and food industries.

Bioprinting is regarded as the one of the future techniques for combining biomaterials, cells, and to some extent, also supporting components into 3D biological constructs to reconstruct deficient tissues or to model tissues and organs in a healthy and diseased state. The goal is to plan the precise positions of cells with computer aided design and then print them individually or layer-by-layer. Bioprinting is a relatively new method and mostly uses biocompatible hydrogels as they allow cell encapsulation in a gelated, hydrated and mechanically supportive 3D environment. In Table 1, a summary of the main processing method highlighting the advantages and the disadvantages of the different preparation methods is presented.

**Figure 11:**
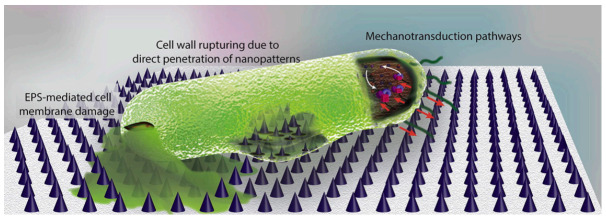
Illustration of the main bactericidal mechanisms of nanopatterns, as described in Reference [150]. While the commonly believed theory is that bacterial cell wall is ruptured by penetration of high aspect ratio nanopatterns, a few studies suggest that EPS plays a key role. It has been shown that the strong attachment of EPS to the nanopatterns and the attempts of bacteria to move away from the unfavorable surface leads to cell membrane damage. Moreover, mechanotransduction pathways in which the mechanical forces affect the metabolomics, and the genomics of bacteria could be possible mechanisms of bacteria death on the surface. (Figure and Figure caption reproduced with permission from Reference [150]).

Chemical modification of surfaces via grafting polymers or copolymers is another well-known approach already mentioned in this review. The grafting of polymers implies chemical modification of the surfaces and the use of specific interactions or steric factors to avoid biofilm formation. Recently, more sophisticated approaches related to surface modification have been reported in some publications [149,150]. Modaresifar and coworkers [150] analyze the use of nanopatterned surfaces to cause the death of bacteria and avoid biofilm development on surfaces. According to these studies, the design parameters of the nanopatterns considered were the height (H), the width and the interspacing between them (iS). The control on these geometrical characteristics is critical so that the surface causes the death of bacteria. Some results suggested that structures with nano-cones with tip diameters less than 20 nm and cone-to-cone interspacings of about 200 nm were able to kill the majority of bacteria [149]. The actual mechanism is not well-known. In some cases, the main mechanism is thought to be due to the mechanical interaction of the cell wall with the nanopattern. However, in some others, it is believed that the extracellular polymeric substance (EPS) plays an important role. The EPS adheres to the nanostructures and when bacteria tries to move away due to the unfavorable interactions with the nanostructures, the EPS anchorage does not let it move, causing a cell wall rupture and cell dead (Figure 11).

The interest of this approach lies on the fact that, if an optimum design of the surface parameters is reached, the use of nanopatterned surfaces would avoid the overuse of antibiotics to kill bacteria. However, although most studies presented in this review [150] report that there were no adverse effects on mammalian cells, one study reported that extremely high aspect ratios (>200 nm) may kill bacteria, but also mammalian cells.

## 4. Characterization Antibacterial Polymer Materials

### 4.1. Microbiological Characterization Methods

In microbiology, the number of viable bacteria in a sample are measured in terms of the colony forming units (CFU), which defines the number of units that can multiply under given conditions. To characterize the effectiveness of a given material against bacterial growth, the number of colony forming units (CFU) in the presence and in the absence of the material is measured. A decrease in the number of CFU (measured in CFU per cm^2^) indicates that the material is effective against a certain strain. In order to evaluate the number of CFU developed on materials after incubation in a culture media with a bacterial strain, Maison et al. [128] used the ASTM E2149-13a standard test method “Determining the antimicrobial activity of immobilized antimicrobial agents under dynamic contact conditions” although some modifications related to sample size and the type of bacteria strains were employed.

**Figure 12:**
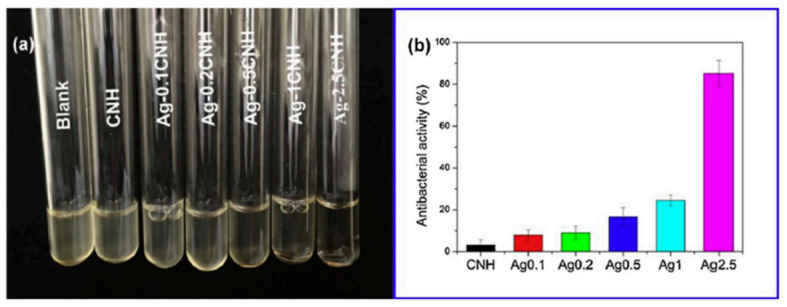
(**a**) Photographs associated to bacterial suspensions of a media containing an *E-Coli* strain co-cultured with chitin-silver nanoparticles, Ag-CNH. (**b**) Results of the evaluation of antibacterial activity obtained after measuring the optical density of the solutions at 600 nm. (Figure reproduced with permission from Reference [154].

Usually, to assess the inhibitory action of material against one or more strains of bacteria one common test is the Kirby-Bauer diffusion test on agar culture. Discs of the material are placed on agar media and cultured for 24 h at 37 °C and the zone of inhibitions (ZOI) formed at the vicinity of the discs are measured after the incubation time [151,152,153]. Another well-known study to evaluate the antibacterial behavior of a material consists in the preparation of a suspension containing a bacterial strain and a co-culture of the strain in contact with the materials to be tested. After the incubation time, the optical density of the solution is measured, and so the efficacy of the material against a given strain can be measured [154]. For example, the results from Song and colleagues [154] showed that the turbidity of the suspension of *E. Coli* decreased with the content in the Ag of the materials tested (Ag-2.5CNH) (Figure 12).

### 4.2. Microscopy Characterization Techniques

Morphological and structural characterization in composite materials is important. For example, in the multicomponent materials used for scaffolds, it is important, among others things, (i) to visualize the distribution of the particles within the polymer matrix; (ii) to determine the porosity of the material, volume fraction of pores, the type of porosity and connectivity and (iii) in fibrous materials, to determine the diameter of fibers and/or the presence of other morphologies such as corpuscles. For all these issues, microscopy characterization techniques are used. While microscopy characterization techniques are also utilized to assess the presence of bacteria on different surfaces upon a bacterial culture.

Microscopy characterization techniques like scanning electron microscopy (SEM), transmission electron microscopy (TEM) and confocal laser scanning microscopy (CLSM) can provide valuable information. The microscopic techniques are used for a double purpose. In polymer nanocomposite materials, filled with antibacterial nanoparticles, it is important to observe how particles are distributed within the polymeric matrix or on the surface of the matrix and whether its distribution is homogenous or not, which will help to understand the in-service behavior of the materials. For that purpose, TEM and SEM (SEM-EDX) measurements are done to observe particle distribution. Sometimes, EDX is used to obtain mappings of the elements in the composite materials, confirming the distribution of the particles within the polymer matrix [155,156].

In addition, the microscopy techniques will give us a complementary morphological and structural analysis to understand and interpret the antibacterial behavior of the composite materials. Using confocal laser scanning microscopy and commercial viability kits, such as the live/dead backlight bacterial viability kit (L-7012, Invitrogen^TM^), many authors test the efficacy of their antibacterial materials. Briefly, this commercial kit contains propidium iodide, a red fluorescent dye, which only penetrates damaged cell membranes. Therefore, it is used to label selectively dead bacterial cells. The other fluorescent dye, commercially known as SYTO^®^9, can interact with all cells and the ones that are intact or damaged membranes and so it is used to label all the cells. According to the suppliers, when cells are incubated with the SYTO^®^9, live bacteria with intact membranes fluoresce are green, while the dead bacteria stain with PI is fluorescent red. This can be explained by considering that PI has a higher affinity for DNA than SYTO9, and therefore PI is able to displace SYTO9 and viable cells are seen as green and dead/damaged cells are red [157]. However, the use of this test has some limitations. First, it depends on the bacteria strain. If the bacteria have intrinsic fluorescence, like in *P. aeuginosa*, this commercial test cannot be used. For other bacteria (*C. albicans* and *yeasts*), different assay kits can be used [158]. A critical review of many practical aspects to be considered when using this kit is presented in Reference [159].

Apart from the antibacterial properties of materials, cell viability studies are done to assess the applicability of the material in biomedicine, food science and technology and any other application involving the contact with cells. Similar experiments to those previously described for bacteria are done and different commercial kits are available. For example, J. González-Benito and colleagues [70] tested the cell viability for EVA40-CuNPs composites with a dye LIVE(calcein)/DEAD(Eth-D-1)^®^ Viability/Cytotoxicity Kit for mammalian cells from (Invitrogen™). These studies revealed the antibacterial properties of CuNps added to poly(ethylene-*co*-vinylacetate) and the viability of HaCat cells.

**Figure 13:**
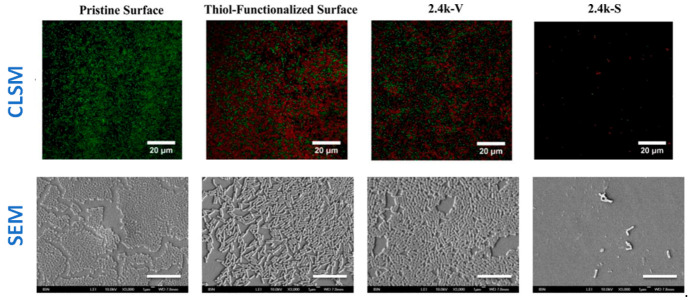
(**Top row**) CLSM micrographs. Images show the results of the Live/dead assay on the uncoated silicone PDMS surface and surfaces coated with thiol, 2.4k V, and 2.4 k-S for *E. Coli* culture after 1 day. The surfaces were imaged under confocal laser scanning microscopy (green denotes live cells; red denotes dead cells; Scale bar = 20 µm). (**Bottom row**) FE-SEM images of *E. coli* after 1 day of incubation on uncoated and coated PDMS surfaces. Size of the scale bar: 10 μm. (Reproduced with permission from Reference [31]).

LCSM is a very versatile technique, which allows for a fast response to assess the antibacterial behavior of materials. However, the visualization of surfaces with other microscopy techniques, such as SEM, can provide important information on bacterial growth and biofilm formation. For example, in a research work by Yang and colleagues [31], SEM micrographs on the surfaces of different samples after one day culture with *E. Coli* strain were done (Figure 13). SEM micrographs showed that a biofilm was formed on pristine, thiol-functionalized and 2.4k-V surfaces, whereas just isolated bacteria were observed in the 2.4 k-S surface. In addition, the live/dead assays were done and LCSM micrographs of the same samples were observed, thus obtaining complementary information on whether bacteria in the biofilms were alive or dead.

**Figure 14:**
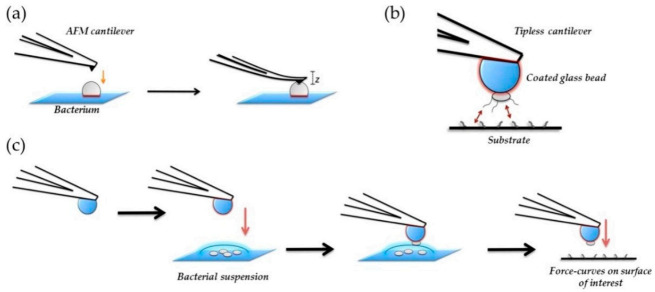
Schematic representation of bacterial nanomechanics experiments for (**a**) nanoindentation and (**b**), and (**c**) single-cell force spectroscopy (SCFS). In nanoindentation Table 2015. NanoTable 26. 062,001 [161].

Atomic force microscopy (AFM) has been demonstrated to be another microscopy technique very useful in the characterization of antibacterial materials: (i) for the visualization of the nanoparticles, (ii) for the examination of particle distribution within the polymer matrices, and (iii) for the morphological characterization of bacterial cells and bacterial colonies, etc. AFM offers a high resolution, allowing us to obtain detailed information in-situ and in air or in aqueous solution important aspects such as the presence of EPS, the quality of the cell wall or the bacterial dimensions without damaging the cell or staining the samples. However, one of the main disadvantages of AFM is the scan size, with an area that is smaller than in SEM, and so, to get an overall view of the general behavior of the whole surface, too many experiments are necessary. For this reason, future trends in AFM evolve in the study of local mechanical properties and specific tip-sample interactions, known as force spectroscopy. In the field of microbiology, one interesting application is the study of mechanical properties of the cell wall by the use of simple indentation modes and cells adhesion by single cell force spectroscopy [90,160]. These kinds of studies are important because mechanical properties of the cell wall may vary depending on the particular effect exerted by the material where the microorganism is adhered. In Figure 14, a scheme illustrating the different kind of mechanical tests that can be done to study mechanical properties of bacteria are presented. 

**Figure 15:**
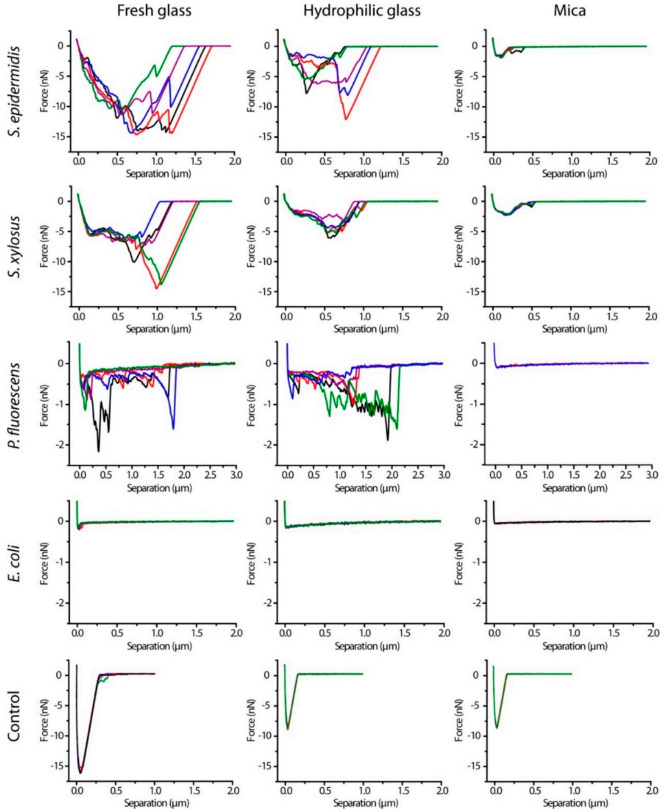
Representative retraction force curves of *S. epidermidis*, *S. xylosus*, *P. fluorescens*, and *E. coli* single-cell probes and control probes (Cell-Tak-coated cantilevers) on three surfaces after contact for 10 s (Figure reproduced with permission from Langmuir 2014, 30, 14, 4019–4025 [162]).

In Figure 15, force curves of Single-Cell Probes of four bacteria on three different surfaces were collected to measure adhesion forces of bacteria. The curves presented in Figure 15 correspond to retraction curves. Adhesion of single-cell probes of four bacterial strains were measured on three different surfaces: fresh glass, hydrophilic glass and mica. A remarkably different mechanical behavior was observed in *E. Coli* compared to the other bacteria.

**Figure 16:**
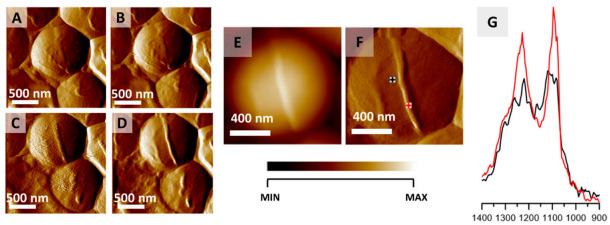
Monitoring *S. aureus* cell division via AFM-IR. (**A**–**D**) AFM images of *S. aureus* cell showing the formation of septum preceding cell division. Size of imaged area: 2 × 2 µm. The images were selected from a larger series (12 images recorded every 20 min) and represent data recorded every 40 min. (**E**,**F**) AFM height and deflection image recorded at the end of cell septum formation with marked points of collection of AFM-IR spectra. Size of the imaged area 1.17 × 1.15 µm. The height of the newly formed structure is 45 nm. (**G**) AFM-IR spectra recorded from cell area (black) and septum area (red) (marked in (**F**)), in the range 1400–900 cm^−1^. Both spectra were normalized to the amide I band and demonstrate an increase in the relative intensity of cell wall components from the septum. This figure is reproduced with permission from Kochan, K., Peleg, A. Y., Heraud, P., Wood, B. R. Atomic Force Microscopy Combined with Infrared Spectroscopy as a Tool to Probe Single Bacterium Chemistry. J. Vis. Exp. (163), e61728, doi:10.3791/61728 (2020) [163].

Another interesting study was done by Kochan and coworkers [163], with the aid of two complementary techniques—AFM and IR spectroscopy. The use of AFM allowed for the visualization of *S. Aureus* bacteria and the division process was observed (Figure 16). The use of IR allowed us to obtain information on the chemical species that take part in the division process, as illustrated in Figure 16G, providing a novel tool for bacterial research, capable of nanoscale probing of the chemical composition.

### 4.3. Surface Characterization

**Figure 17:**
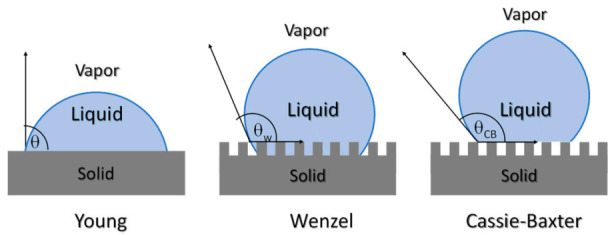
Scheme illustrating the different models used to interpret wettability behavior Young, Wenzel and Cassie-Baxter.

The characterization of surface properties is essential, particularly from the point of view of the preparation of materials with micro or nanopatterned surfaces, as one alternative to prepare antifouling structures. The study of surface properties of materials can be addressed using the main characterization techniques: (i) surface roughness and (ii) contact angle measurements. The characterization of surface roughness can be addressed, at the microscale, with the aid of a profilometer, or at the nanoscale, with the use of an atomic force microscope. The parameters frequently used to measure surface roughness are, Rp; maximum height Rz; arithmetic mean height, Ra; and root mean square height, Rq. The surface roughness can influence the type of bacteria and the amount of bacteria that adhere to the surfaces [164]. Apart from roughness, the wettability of surfaces also can affect the bacterial growth and biofilm development on surfaces. Surface roughness and physico-chemical characteristics can lead to the formation of antifouling surfaces. Surface wettability plays an important role in many physical, chemical and biological processes. Recently the study of contact angle measurements has also become important for the comprehension of permeation and antifouling properties of membranes. The sessile-drop method is one of the most used, due to its simplicity, although to avoid obtaining misleading results, careful measurements should be done, to calculate the surface energy [165]. The wetting characteristics of a surface in water (or other liquids) can vary due changes in the composition, for example with the content in nanoparticles or with changes in surface topography. In Figure 17, different methods to interpret the wettability and the liquid-solid interactions such as Young, Wenzel or the Cassie-Baxter model. The Cassie-Baxter model explains the formation of superhydrophobic surfaces due to a minimum contact surface. The so-called Lotus effect is one example of such behavior. The microstructure of the Lotus leaf does not allow for water to penetrate, leading to self-cleaning surfaces and thus lowering the bacterial growth. Therefore, the nano and microstructure of a polymeric substrate can lead to superhydrophobic surfaces with antifouling properties [135].

## 5. Future Perspectives

Infections caused by bacteria are the cause of fatal diseases. The research and development of novel multifunctional polymer-based materials with antibacterial properties is therefore essential. Considering the complex nature of this problem, future perspectives will probably go in the direction of combined approaches, studying different methodologies, such as the development of nanopatterned and nanostructured surfaces with controlled surface properties and chemical composition to minimize bacterial adhesion. This considers the use of antifouling materials such as PEG or polycarbonates, combined with the incorporation of active nanoparticles and the use of a particular processing method to obtain controlled nanostructured surfaces.

## Figures and Tables

**Table 1:** Summary of the main processing methods, advantages and disadvantages.

Processing Method	Advantages	Disadvantages
Melt processing	Scalable to industry Well-known method With high content of particles, aggregates can form	Active molecules sensitive to temperature can degrade or oxidize
Solvent casting	Easy to implement Laboratory scale (Petri dish)	Difficult to scale up for large productions Compositional gradient due to gravity in composites May need a post-processing (hot-pressing stage) Not good for polyolefins
Electrospinning	Controlled morphology of the fibers and fiber size Controlled orientation of the fibers due to the electrical field Good for finishing treatments Allows obtaining surface micro- or nanostructure	The polymer and the solvent should be polar (electrical conductivity) For high concentration of particles or particles with big particle size, the nozzle can clog
Solution Blow Spinning	Versatile method that can be used with most polymers Good for applying finishing treatments Allows obtaining surface micro- or nanostructure to control antifouling/antimicrobial properties	For high concentration of particles or particles with big particle size, the nozzle can clog
Solid state methods (High Energy Ball Milling, HEBM)	Good dispersion of particles even for high loadings Can be used in industry Good results for food industry	May cause chain scission of the polymers or degradation of the active molecules. If not optimized properly, may cause cross-contamination
Sputtering	Good for finishing treatments Easy with metals Only Nylon and Teflon have been reported	Metals can migrate and antibacterial action is lost. Difficult to implement for polymers (chain scission or crosslinking reactions).
Plasma Treatment	Useful to produce locally antibacterial surfaces	Local treatment, it is not possible to
Nanopatterned surfaces	Highly specific Avoid the overuse of antibiotics	Requires further investigation on the aspect ratios of the nanoparterns to kill each bacterium. Investigation on cell viability is also needed.

## Data Availability

Not applicable. All data are available in the review and references therein.

## Abbreviations

AAS	atomic absorption spectroscopy
AFM	atomic force microscopy
AgNPs	Silver nanoparticles
BTE	bone tissue engineering
CAUTI	Catheter-associated urinary tract infection
CLSM	Confocal Laser Scanning Microscopy
DRI	Device related infections
CNTs	Carbon nanotubes
CV	Crystal Violet
EPS	Extracellular Polymeric Substance
HEBM	high energy ball milling
MDR	Multidrug resistant bacteria
MRSA	Methicillin resistant Staphylococcus Aureous
MWCNTs	Multiwall carbon nanotubes
LDPE	Low Density Polyethylene
nD	nanodiamond (nD)
nHA	nanohydroxyapatite
PCL	poly(ε-caprolactone)
PEG	polyethylene glycol
PET	Polyethylene terephthalate
PGA	polyglycolic acid
PGS	poly(glycerol sebacate)
PHB	polyhydroxybutyrate
PHBV	Poly(3-hydroxybutyrate-co-3-6%hydroxyvalerate)
PLA	polylactic acid
PLGA	polylactic-co-glycolic acid
PVDF	Poly(vinylidene fluoride)
QCSP	Quaternized chitosan-g-polyaniline
QDs	Quantum Dots
ROS	Reactive Oxygen Species
SEM	Scanning Electron Microscopy
SSPB	Siloxane sulfopropylbetaine
SWCNT	Single walled carbon nanotubes
TE	Tissue Engineering
TEM	Transmission Electron Microscopy
WHO	World Health Organization
